# Analysis of the Dielectric Constant of Saline-Alkali Soils and the Effect on Radar Backscattering Coefficient: A Case Study of Soda Alkaline Saline Soils in Western Jilin Province Using RADARSAT-2 Data

**DOI:** 10.1155/2014/563015

**Published:** 2014-07-01

**Authors:** Yang-yang Li, Kai Zhao, Jian-hua Ren, Yan-ling Ding, Li-li Wu

**Affiliations:** ^1^Northeast Institute of Geography and Agroecology, Chinese Academy of Sciences, Changchun 130102, China; ^2^Graduate University of the Chinese Academy of Sciences, Beijing 100049, China

## Abstract

Soil salinity is a global problem, especially in developing countries, which affects the environment and productivity of agriculture areas. Salt has a significant effect on the complex dielectric constant of wet soil. However, there is no suitable model to describe the variation in the backscattering coefficient due to changes in soil salinity content. The purpose of this paper is to use backscattering models to understand behaviors of the backscattering coefficient in saline soils based on the analysis of its dielectric constant. The effects of moisture and salinity on the dielectric constant by combined Dobson mixing model and seawater dielectric constant model are analyzed, and the backscattering coefficient is then simulated using the AIEM. Simultaneously, laboratory measurements were performed on ground samples. The frequency effect of the laboratory results was not the same as the simulated results. The frequency dependence of the ionic conductivity of an electrolyte solution is influenced by the ion's components. Finally, the simulated backscattering coefficients measured from the dielectric constant with the AIEM were analyzed using the extracted backscattering coefficient from the RADARSAT-2 image. The results show that RADARSAT-2 is potentially able to measure soil salinity; however, the mixed pixel problem needs to be more thoroughly considered.

## 1. Introduction

Soil salinization is a global environmental problem with severe economic and social consequences, especially in arid and semiarid regions. It is one of the main causes of land degradation and productivity loss. Almost 3% of the world's soil resources, the equivalent of 4.025 million hectares (Mha), are salt-affected due to varying degrees of human-induced degradation [1]. Thus, the monitoring of the area and degree of land salinization is of important value to agriculture.

Radar remote sensing is known to be sensitive to several natural surface parameters such as surface roughness, vegetation, and soil moisture. Until now, no inversion algorithms existed for directly determining the soil salinity content from microwave remote sensing data. However, the measurement of soil moisture by radar systems has been widely investigated and applied over the last two decades [2–6]. The basic theory of the soil moisture inversion method is that, on bare surfaces, the dielectric constant is highly dependent on soil moisture due to the large difference in the dielectric constant between dry soil (approximately 2-3) and water (approximately 81). As the moisture of dry soil increases, the dielectric constant consequently increases, which directly affects the backscattering coefficient. The complex dielectric constant is composed of two parts, the real part and the imaginary part. The real part (*ε*′) of the complex dielectric constant is related to the medium polarization and governs the velocity of propagation of a wave trough the material, which is called the permittivity; the imagery part (*ε*′′) is related to the conductivity of the medium and represents the radar signal attenuation by energy absorption required to reach the polarization, which is called the loss factor according to Lasne et al. [7]. When comparing the complex dielectric constant of pure water with saline (sea) water (*≅*49.69 mS/cm) [8], a minimal difference is observed in the real part, but there is a significant difference in the imaginary part especially at microwave frequencies less than *≅*7 GHz [9]. Taylor applied the small perturbation model (SPM) to the Pyramid Hill area when the surface soils were fully saturated. A study by Taylor et al. [10] showed that variations in the dielectric constant beyond volumetric soil moisture content of 30% were attributed to changes in the imaginary part. Using RADARSAT-1 SAR data and the laboratory analysis of the dielectric constant with different salt contents, Shao et al. [11] showed that the backscattering coefficient of salt-affected soil is more correlated with *ε*′′ than with *ε*′. Use of radar data, which exhibit sensitivity to the dielectric and geometric characteristics of objects, weather independent imaging capability and potential to acquire subsurface information, is one of the most promising approaches for salt-affected soils, practical for high soil moisture content conditions. However, there are rare theoretical and empirical backscattering models to describe the relationship between the dielectric constant, soil salinity, soil moisture content, and the backscattering coefficient. The backscattering model used in soil moisture retrieval algorithms can also be used to determine soil salinity.

Based on the work by Aly et al. [13], a theoretical model, AIEM (advanced integral equation model), was used to simulate salinity's effect on the backscattering coefficient of soils, and then the dielectric constant of laboratory measurements compared to RADARSAT-2 data was analyzed based on the theoretical simulation. In this paper, the Dobson mixing model combined with a seawater dielectric constant model is used to simulate the salt-affected soil dielectric constant, whose advantages and disadvantages are then analyzed.

## 2. Study Areas and Measurements

### 2.1. Location

**Figure 1:**
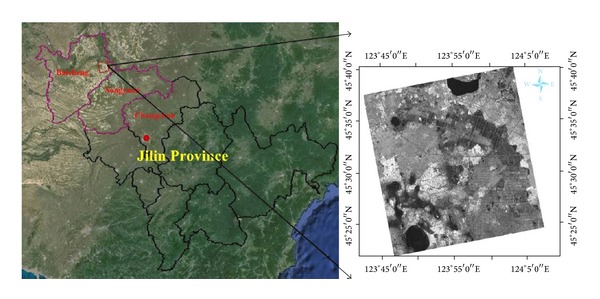
Location of the study area (the image on the right is the HH polarization of RADARSAT-2).

The study site is in An'guang [45°20′–45°40N, 123°42′–124°8′E] of Da'an, western Jilin Province, an area with soda alkali-saline soil shown in Figure 1. The city of Da'an is located in the hinterland of the Songnen Plain. The development of the saline-alkali soil is a comprehensive result of several natural environmental factors, including climate, geology, land formations, parent material, hydrological conditions, water chemistry, and the freeze-thaw factor. The climatic zone ranges from temperate semihumid to semiarid. The evaporative capacity is greater than precipitation and the continental climate is obvious. Spring and autumn are the periods of salification, whereas summer is the salt leaching period, and a frozen layer appears in the winter. Each climatic condition contributes to salinization. The weathering of aluminosilicate magmatic rock contained within the Daxinganling, Xiaoxing'anling, and Changbai Mountains resulted in calcium, magnesium, potassium, and sodium bicarbonate. Then, the dissolved salt in surface water and groundwater concentrated in the plains or low-lying areas, which brought a rich source of soda salt substances. Heavy-textured soil parent material produced by the quaternary geological environment combined with poor drainage and a low local groundwater level formed one of the world's largest soda saline-alkali areas.

### 2.2. Available Data

A RADARSAT-2 SAR image from June 28, 2013, was obtained for the study area in the Fine-Quad-Pol mode with an incident angle of approximately 39°–41°. The primary information of the SAR dataset is shown in Table 1. Before using the radar data, radiation calibration, filter processing, and a geometric correction should be performed first.

Coherent processing occurs from the RADARSAT-2 sensors, which produces a lot of speckle noise. Noise removal should be performed. An enhanced Frost filter with 5 × 5 windows was used to reduce the effect of noise, achieving a satisfactory result. Noteworthy is that the image was taken immediately after a heavy rain event and most of the land surface was fully saturated; therefore, it is advantageous to analyze the salt content from the radar image according to the work by Taylor et al. [10].

**Figure 2:**
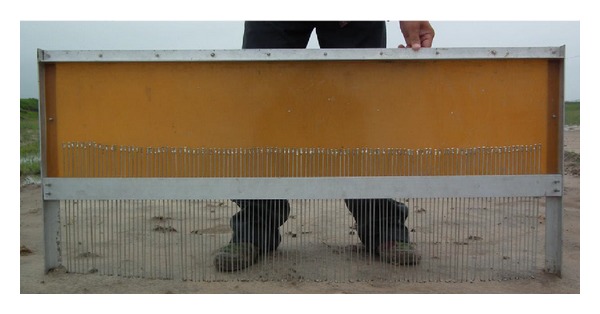
Measurement of surface roughness.

Ground sampling data were collected coincidentally with the RADARSAT-2 overpass on June 28, 2013. 53 samples were collected at the soil surface with a depth of 0–5 cm according to the geomorphologic and pedological characteristics of the area. Real-time soil-moisture, ground EC and the temperature were simultaneously measured using a WET sensor. The average moisture value was 35 ± 10 (%). For the 53 soil samples, details from 17 points were taken for laboratory dielectric constant analysis using a vectorial network analyzer. Surface roughness was measured manually using a 1.5-m-long pin profiler shown in Figure 2, and there were at least 3 repeat measurements of each point. Most sample points of bare ground in the region were of smooth surfaces. The average value of the RMS (root mean square) height (*s*) was 0.27 cm, and the correlation length (*l*) was 3.5 cm.

## 3. Methodology

### 3.1. Soil Dielectric Mixing Models

The influence of soil saline deposits in microwave remote sensing is related to the solubility behavior and the ionic properties of the minerals. In the ionic form, the weight of dissolved substances per kilogram of solution determines the conductivity of the solution; that is, the conductivity is directly related to the presence of free electrons and ions. Under the function of an extra electric field, the distribution of charges is distorted and the barycenters of positive and negative charges no longer coincide, which results in an induced dipole moment referred to as the polarization vector** P**. There are three types of material in the natural world: atoms, molecules, and ions. The presence of such components generates three microscopic polarization mechanisms: electronic, ionic, and orientation polarizabilities, which are related directly to the medium dielectric properties. More importantly is that *ε*′′ is proportional to the conductivity. Because the propagation of the radar signal is primarily governed by the dielectric constant, it is necessary to understand the behavior of both the real and imaginary parts of the dielectric constant with the increase in soil salinity.

Theoretical, semiempirical, and empirical mixture models were proposed for determining the dielectric constant of soil materials, such as the model by Wang and Schmugge [14]. Soil not affected by salt is usually considered to be a mixture of four components: soil, air, free water, and bound water. To describe the dielectric constant of such a mixture, Dobson et al. [12] developed a semiempirical model for soil. Given a bulk density *ρ*_*b*_ and specific density *ρ*_*s*_, the model is described as

(1) $$\begin{array}{c} \varepsilon_{m}^{\alpha }=1+\frac{\rho_{b}}{\rho_{s}}\left(\varepsilon_{s}^{\alpha }-1\right)+m_{v}^{\beta }\varepsilon_{fw}^{\alpha }-m_{v}, \end{array}$$

where *α* = 0.65, *ε*_*s*_ = (1.01 + 0.44*ρ*_*s*_)^2^ − 0.062 is the dielectric constant of soil particles, *β* is a coefficient expressed as a function of sand and clay contents, *m*_*v*_ is the volumetric soil moisture content, and *ε*_*fw*_^*α*^ is the dielectric constant of the free water.

The electric conductivity of the saturated soil sample used in the Dobson model is approximately 1 dS/m, which was considered to be nonsalt-affected soil. The criterion between salt-affected and nonsalt-affected soil is 4 dS /m; thus, the model is not suitable for the salt-affected soil. To accurately describe the dielectric constant of salt-affected soils, Aly et al. [13] replaced the dielectric constant of free water with Stogryn's saline water model [8], the same method that we also used in this paper. The soil surface temperature is fixed at 23°C, which is acceptable for an average value of sampling soils. The bulk density used in the Dobson model is 1.4 g/cm^3^, there was an average value of 53 ground points, and the specific density was assumed to be 2.65 g/cm^3^.

### 3.2. Backscattering Model

To evaluate the effect of salts on airborne or orbital SAR data, the radar backscattering coefficients based on the model need to be computed for the dielectric constant values previously simulated.

The backscattering models used in nonsalt-affected soils are also suitable for salt-affected soils. There are several classic backscattering models of random rough surfaces proposed in previous research (theoretical and empirical models), such as the SPM (small perturbation model), the POM (physical optic model) introduced by Ulaby et al. in microwave remote sensing [15], the IEM (integral equation model) and AIEM (advanced integral equation model) proposed by Fung et al. [16, 17], and the DM (Dubois model) proposed by Dubois et al. [18]. Different models have different application fields.  Table 2 shows the applicability of these models.

The SPM assumes that variations in surface height are small relative to the wavelength and that the incidence angle is greater than 35°–40°. The POM is valid when the RMS surface slope is small relative to the wavelength and the incidence angle is smaller than 35°. The Dubois model is suitable for frequencies between 1.5 GHz and 11 GHz. Contrary to the three models, the model based on the surface integral equation (IEM/AIEM) is the most suitable model for all surface types.

This approach was reported by Fung and Chen [16], where the phase of Green's function in a higher order term was ignored. This phase was later retained by Hsieh and Fung by fully including the phase effect in Green's function to achieve better accuracy in bistatic scattering and multiple surface scattering, especially in regions where the incident and scattering angles are not equal, which was primarily the case for the AIEM. Therefore, we applied the AIEM to simulate the single backscattering coefficient of salt-affected soil by only considering the surface scattering term. The single backscattering coefficient of the AIEM model is expressed as

(2) σpp0=k24πexp⁡(−4kz2σ2) ×{|2kzσ+σ4(Fpp1+Fpp2)|2w(2kx,0)   +∑n=2∞|(2kzσ)nfpp+σ4Fpp1(2kzσ)n−1|2   ×w(n)(2kx,0)n!},

where *p* = *v* or *h* polarization, *θ* is the incident angle, *k*_*x*_ = *k*sin*θ*, *k*_*z*_ = *k*cos⁡*θ*, *f*_*vv*_ = 2*R*_*v*_/cos⁡*θ*, and *f*_*hh*_ = 2*R*_*h*_/cos⁡*θ*. The expressions *w* and *w* ^(*n*)^ are the surface spectra corresponding to the two-dimensional Fourier transformations of the surface correlation coefficient and its *n*th power.

It is apparent that most naturally occurring surfaces, land or sea, may contain more than one scale of roughness. Exponential statistical distribution and Gaussian and the *x*-power law distribution can all be used to describe the natural surface as described by Fung and Chen [16] who also showed that, for moderately rough surfaces, an exponential statistical distribution performs better than the Gaussian or the *x*-power law distributions (*x* = 1.5). Considering the relatively smooth surface of saline soil, the exponential statistical distribution was chosen for describing the surface of the AIEM model [19, 20].

The incident angle used in the model is assumed to be 40°, comparing the incident angles of the RADARSAT-2 image.

### 3.3. Laboratory Measurement of the Dielectric Constant

To determine the validity of the previous dielectric mixing model and simultaneously assess the impact of salt on the RADARSAT-2 data, we performed dielectric constant measurements for 17 samples with different gravimetric water contents (from dry soil to 50% with a step of 5%). The volumetric soil moisture was measured using the oven-drying method. A vectorial network analyzer (Agilent 83630A PNA Analyzer) coupled with an open-ended coaxial probe technique was used in the measurement of the soil dielectric constant [7, 11]. The measurement frequency was from 0.5 GHz to 40 GHz with 200 sampling points.

Because the electromagnetic field at the probe/sample interface can be represented by a capacitor, a calibration procedure had to be performed on which the dielectric constant and frequency are well known. The dielectric constant measurement system was calibrated using a standard calibration procedure provided by the system (air-short circuiter-distilled water), and we refreshed the system before every sample was measured.

## 4. Results

### 4.1. Analysis of the Simulated Dielectric Constants

The soil soluble salt in the groundwater can rise to the surface as water moves through capillary action in the soil. Then the water evaporates, resulting in salt accumulation, which forms saline soils. The particle size of different soil textures can affect the speed and height of water movement through capillary action in the soil. In general, the capillary water in loamy soil rises relatively quicker and to a greater height compared to clay and sandy soils. The five different soil texture types were all analyzed, and the representative texture types of this region are sandy and clay soils, which were thoroughly analyzed at three frequencies: L band (1.4 GHz), C band (5.4 GHz), and X band (10.65 GHz).

#### 4.1.1. Salinity and Moisture's Effect on the Dielectric Constant

**Figure 3:**
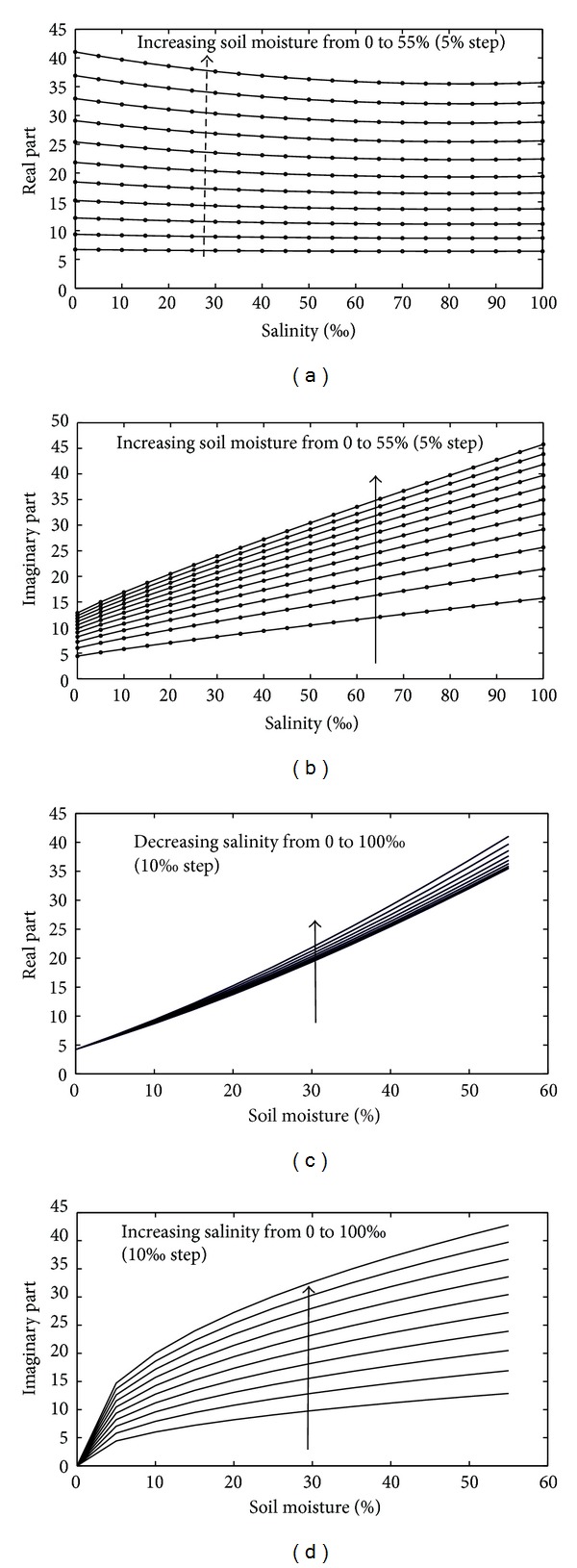
Effect of salinity and moisture on the sandy loam soil dielectric constant derived from the Dobson model combined with the saltwater model at 5.4 GHz. (a) Salinity effect on the real part, (b) salinity effect on the imaginary part, (c) soil moisture effect on the real part, and (d) soil moisture effect on the imaginary part.

Below 10 GHz, the ionic conductivity of saline water may have a marked effect on the loss factor. Consequently, high soil salinity may significantly influence the dielectric properties of wet soil. In this paper, we take sandy loam soil with a frequency of 5.4 GHz as an example. The results in Figures 3(c) and 3(d) show that both the real and imaginary parts increase sharply with the moisture content; however, the impact of salinity is not the same for *ε*′ and *ε*′′. For the real part, the results presented in Figure 3(a) show that soil salinity has little influence on *ε*′. As moisture increases, the effect of salinity increases and the *ε*′ decreases with increasing salinity. Conversely, the imaginary part *ε*′′ is intensely affected by both the salinity and soil moisture. The higher the moisture, the stronger the effect of salinity as shown in Figures 3(b) and 3(d). The imaginary part of *ε*′′ is related to the conductivity of the medium and is generally proportional to the conductivity. The behavior of *ε*′′ increasing with increasing soil moisture can be explained by the higher water content (especially the content of free water) within the soil leading to a greater amount of salts dissolved in the soil system (soil, free water, air, dissolved salt, and bound water), resulting in an increase in conductivity. More precisely, for soil with small water content, the dominant form is bound water in soil with dielectric properties close to ice (approximately 3.2), which leads to a weak increase in the dielectric constant. For soil with greater water content, the free water component in the soil becomes much more active and allows more salt to be dissolved in the soil.

#### 4.1.2. Texture Effect on the Dielectric Constant

Soil texture is one of soil's physical properties. It refers to the different combinations of different mineral particle sizes. Soil texture has a close relationship with soil aeration, the protection of soil fertilizer, water status, and farming ease; therefore, it cannot be neglected when analyzing the dielectric constant of soils. Soil texture affects the detection of soil moisture because the dielectric constant changes with the relative amount of sand, silt, and clay in the soil [12, 14]. In saline soil, the texture can affect salt and moisture's ability to move, which can directly influence the dielectric constant of salt-affected soils.

**Figure 4:**
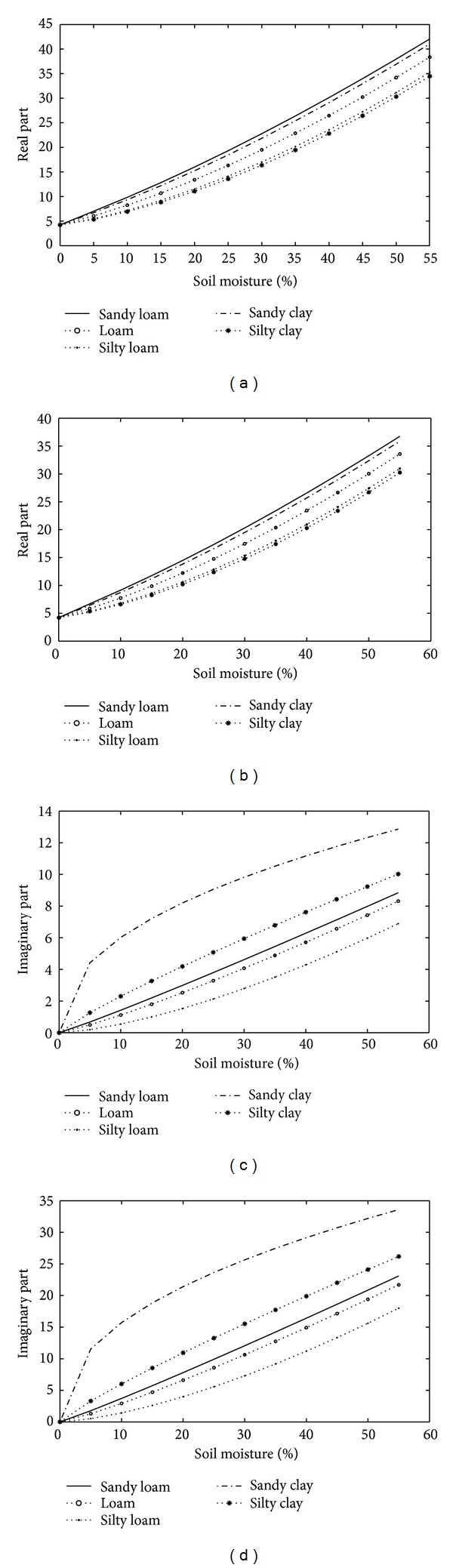
Effect of soil texture on the dielectric constant in nonsalt-affected soil (on the left) [12] and 60‰ in salt-affected soil (on the right). (a) Texture effect on real part (0‰), (b) texture effect on real part (60‰), (c) texture effect on imaginary part (0‰), and (d) texture effect on imaginary part (60‰).

The five different soil texture types were analyzed, and the results are presented in Figure 4. For the oven-dried soil, both the *ε*′ and *ε*′′ are approximately the same for all soil types. The variation of the soil dielectric constant with increasing soil moisture (*m*_*v*_) can be divided into two parts: (i) between *m*_*v*_ = 0 and a transition moisture level *m*_*t*_ and (ii) *m*_*v*_ ≥ *m*_*t*_. The transition moisture is a constant for a given type of soil composition and varies between 0.03 for sands and 0.1 for clays, which represents the boundary between the bound water and the free water molecules [14]. Between the dry soil and the transition moisture level, most of the water molecules in the soil system are considered to be at least partially bound to the soil particles by the influence of both metrics and osmotic forces. Because of the much smaller dielectric constant of bound water molecules compared to free water, consequently the dielectric constant of the mixture increases only slowly with increasing *m*_*v*_. Beyond the transition moisture level, the water molecules are considered to be free particles with a larger dielectric constant than that of dry soil, and thereby a strong influence occurs on the soil system. The transition moisture depends on the soil particle surface area per unit volume and is a function of soil type. Sandy loam soils have the highest *ε*′ for both salt-affected soil and nonaffected soil, followed by sandy clay and then silt clay. The results show that the higher the percentage of sand content in soil particles, the higher the real part of the soil dielectric constant. This is the reason that the sand has a relatively weak ability to bind the water molecules to soil particles leaving more free water in the soil system, which results in a higher dielectric constant.

The effect of texture on the dielectric loss (*ε*′′) is more complicated than the real part. The *ε*′′ is shown to increase with soil clay content. For nonsalt affected soil, at 4.0 to 6.0 GHz, *ε*′′ is nearly independent of soil texture at all soil moisture conditions as proposed by Schmugge (1983). However, for saline soil, the effect of texture at 5.4 GHz is strong. We also confirm that the texture effect increases with an increasing salinity content.

#### 4.1.3. Frequency Effect on the Dielectric Constant

The dielectric loss *ε*′′ is a parameter that describes the movements of electric charge (conduction phenomena). The conduction can arise from an actual transport charge (such as ionic conduction in electrolytes). Thus, the observed dielectric loss consists of two terms—the loss due to a lag in polarization and the conductive loss. Both of the terms depend on the frequency, especially below 10 GHz where the ionic conductivity of saline water may have a marked effect on the loss factor. Thus, the aim is to assess the frequency effect on the dielectric constant. Four commonly used microwave frequency bands were analyzed—L (1.4 GHz), S (2.4 GHz), C (5.4 GHz), and X (10.65 GHz).

**Figure 5:**
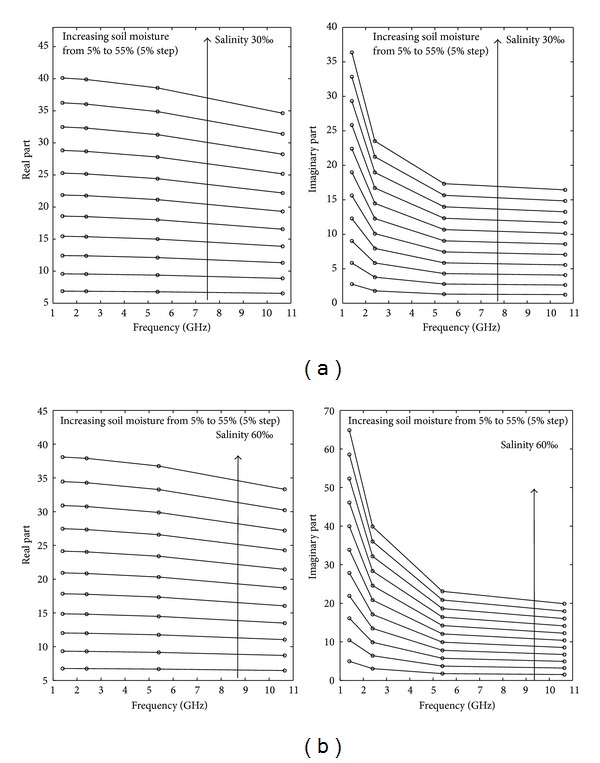
Effect of frequency on the dielectric constant at a salinity of 30‰ (a) and 60‰ (b).

**Figure 6:**
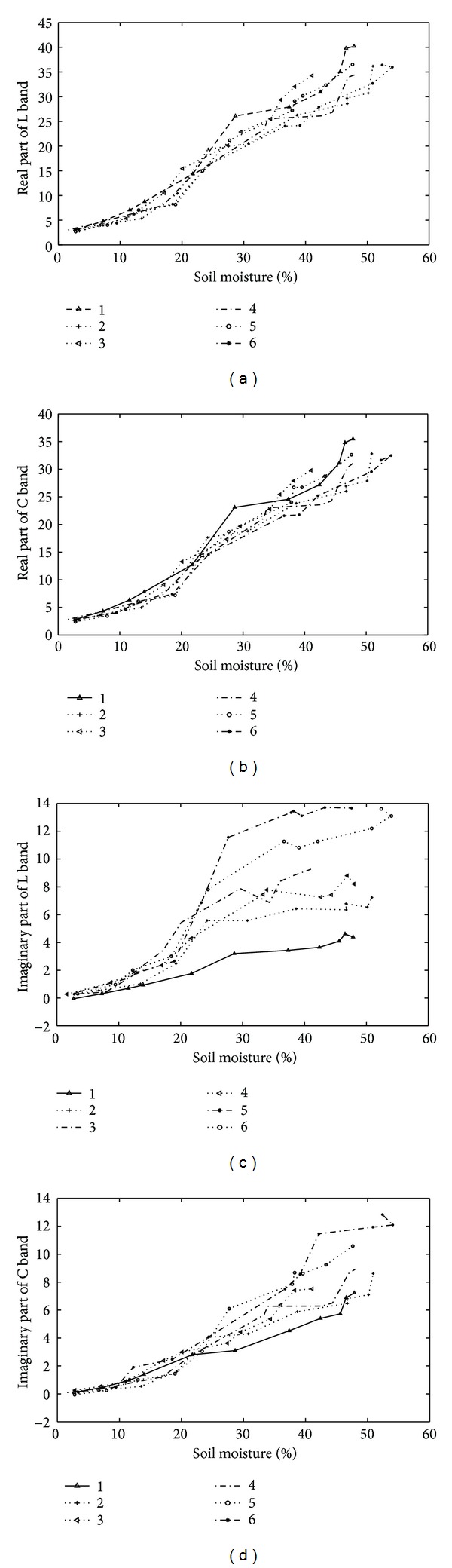
Measurement of the dielectric constant of the L band and C band (6 sample points are taken with different salinity contents as an example).

The frequency variation of the dielectric constant of salt-affected soils is shown in Figure 5 (salinity of 30‰) and Figure 6 (salinity of 60‰). The volumetric water content is in the range of 5% to 55%. The real part *ε*′ slowly decreases with an increase of frequency. Conversely, the imaginary part *ε*′′ rapidly decreases with an increase of frequency, especially in the low frequency range; the value stabilizes when the frequency is higher than 7 GHz. Furthermore, Shao achieved the same *ε*′′ variation pattern from experimental measurements performed on NaCl-concentrated solutions, and the results from Lasne's combination of the Wang model and seawater dielectric model to simulate the salt-affected soil dielectric constant confirmed our study's conclusion.

### 4.2. Analysis of the Laboratory Measured Dielectric Constant

Six samples of different salinity content were chosen and the dielectric constant was then analyzed in the L band (1.43 GHz) and C band (5.4 GHz). The results are shown in Figure 6. The salinity contents were increased from sample 1 to 6 in turn. For the real part, both the L and C bands increased with the increasing water content, and the values in the L band were slightly greater than the C band. In addition, there was a higher salinity content in the lower real part, as shown in Figure 6, especially when the water content was greater than 20%. This phenomenon was the same as the model's simulated result, as presented in the previous section. The imaginary part increased with the increasing water content; however, when the water content was greater than 20%, the influence of salinity on *ε*′′ leads to a strong increase. However, in instances of low volumetric water content, the salinity effect is unnoticeable for both the real and imaginary parts of the dielectric constant. This is because the low volumetric water content results in a low concentration of ions (namely, conductivity). The results shown in Figure 6 also indicate that the C and L bands are highly correlated, especially for the real part. The research results of Shao showed that the C and L bands were favorable for delineating the soil salinity.

**Figure 7:**
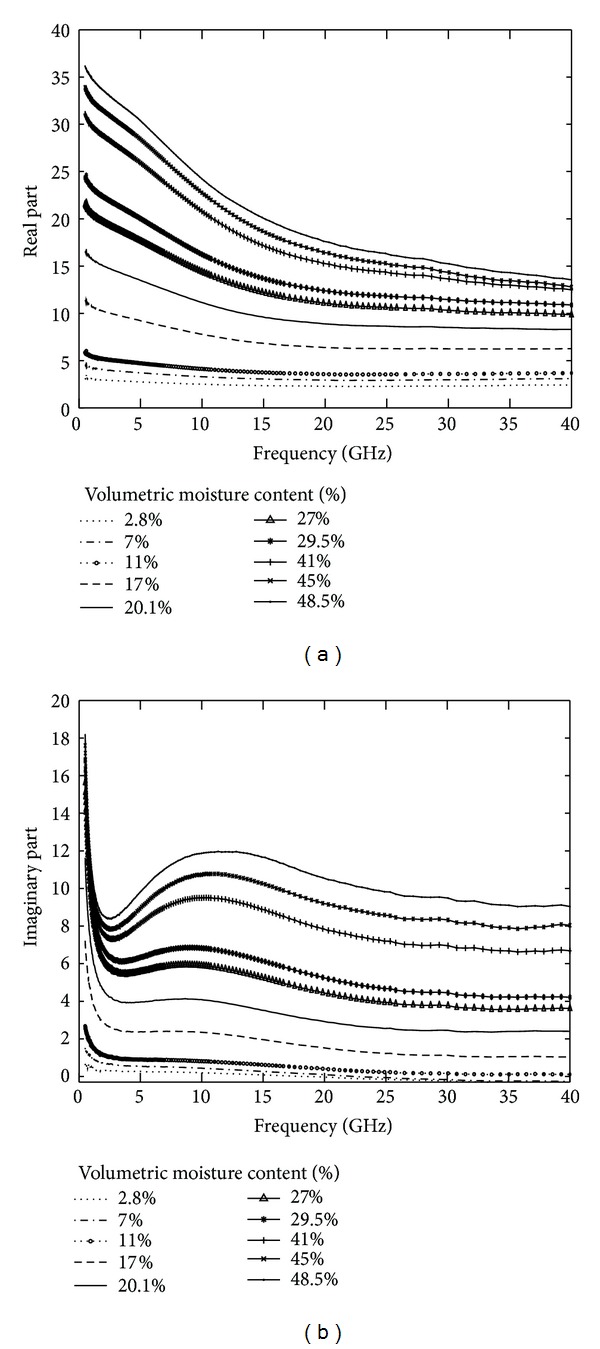
Frequency variations of the real (a) and imaginary (b) parts of the dielectric constant of the sample derived from laboratory measurements at different moisture content levels.

The measurement results of the sample prepared in the laboratory were compared with the frequency variation as shown in Figure 7. The real part decreased with the increasing frequency, especially in instances of high water content where the real part decreased sharply with a variation in frequency. The measured results of the frequency effect on *ε*′ were the same according to the results of the mixed model (the Dobson model combined with the seawater dielectric constant model). However, a distinct phenomenon occurred with the imaginary part. At a low frequency (0.5–2 GHz), the *ε*′′ decreased sharply with an increasing frequency, which confirmed the theoretical frequency dependence of *ε*′′, exhibiting a steep variation rate in the 1-2 GHz frequency range [7, 11]. Then, an inflection point appeared, after which the *ε*′′ increased with an increasing frequency from 3 GHz to 10 GHz. At frequencies greater than 10 GHz, the *ε*′′ began to decrease. This phenomenon was present in all of the measurement samples prepared in the laboratory. When the frequency was higher than 5 GHz, the measured results were not the same as the research of Lasne et al. [7] and Shao et al. [11]. An explanation for the different conclusions follows.

The laboratory research of both Shao and Lasne was based on a mixture of an NaCl solution and soil, and the salinity water model used in Section 4.1 was a seawater model, which is also based on the analysis of NaCl solution; however, the salt components for this study were Na^+^, CO_3_^2−^, and HCO_3_^−^. In an electrical field, the process of a medium changing from a state of nonpolarization to polarization or changing from one polarization state to another is called the electric polarization relaxation process, and the time of the relaxation process is the dielectric relaxation time. An investigation was performed by Chandra and Bagchi [21] that showed the saturation of a solution might be attributed to the influence of the relaxation time on the saturated solution. In solutions, the variations of *ε*′′ with the frequency can be illustrated as the frequency dependence of ionic conductivity, which usually describes the motion ions [22]. Different salt components have different ionic conductivity properties, which results in different relaxation times that then influence the frequency. The relaxation time is also related to the solution temperature and the concentration.

The results presented in Figure 7 show the saline soil in the study area by combining the C band (5.4 GHz) and X band (10.65 GHz). This conclusion also confirmed that combining the Dobson model with the seawater dielectric constant model was not suitable for all types of salt-affected soil. It is necessary to develop suitable models for different saline-alkali lands.

### 4.3. Analysis of the Simulated Backscattering Coefficients Based on the Simulated Dielectric Constant

**Figure 8:**
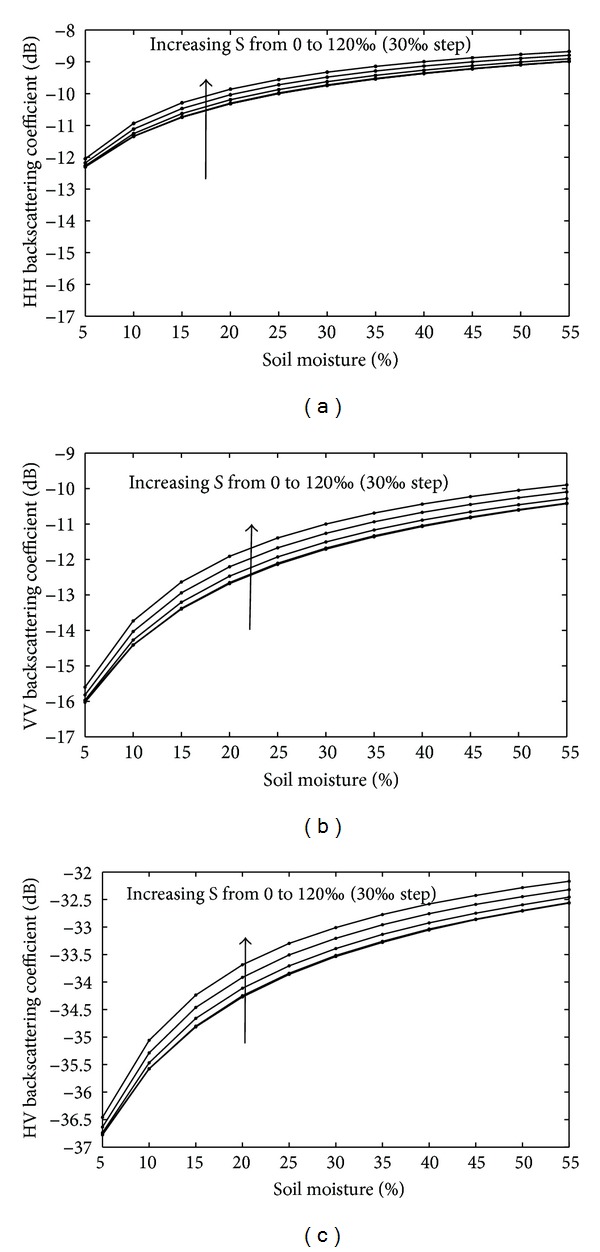
AIEM simulations of the simulated dielectric constant for the backscattering coefficient at a C band of 40° incidence angle with a variation in soil moisture and soil salinity. (a) HH polarization, (b) VV polarization, and (c) HV polarization.

The backscattering coefficient model used in the paper is the AIEM model, which was introduced in Section 3.2, and the band is 5.4 GHz, referred to in the RADARSAT-2 data. The input dielectric constant data were previously simulated by combining the Dobson model and seawater model. The surfaces of the salt-affected soils are usually smooth. Thus, an RMS height of 0.27 cm and a correlation length of 3.5 cm were set for the data simulation. The simulated results in Figure 8 show that the sensitivity of the backscattering coefficient on the soil's salinity depends on the soil moisture content. With an increase in soil moisture content, the simulated backscattering coefficient increased, which was the same effect on salinity. Salinity more slightly affects the HH polarization mode compared to the other two simulated modes, particularly where the soil moisture content is high. For HH polarization, the range of variation is −12 dB to −9 dB, whereas the VV polarization is from −16.2 dB to −10 dB and from −37.3 dB to −32.2 dB for HV polarization.

There may be two possible explanations for interpreting these characteristics. The first is that soil moisture greatly dominates the dielectric constant for soils with high moisture content; therefore, when the salinity increases, the change in the backscattering coefficient is not obvious. The second explanation may be that the salt in the soil cannot be completely dissolved at a low water content—the greater the water content, the more the free ions in the soil system and, subsequently, the higher the dielectric constant. When the dielectric constant is high enough, the Fresnel coefficients change slowly, which is due to radar signal saturation.

**Figure 9:**
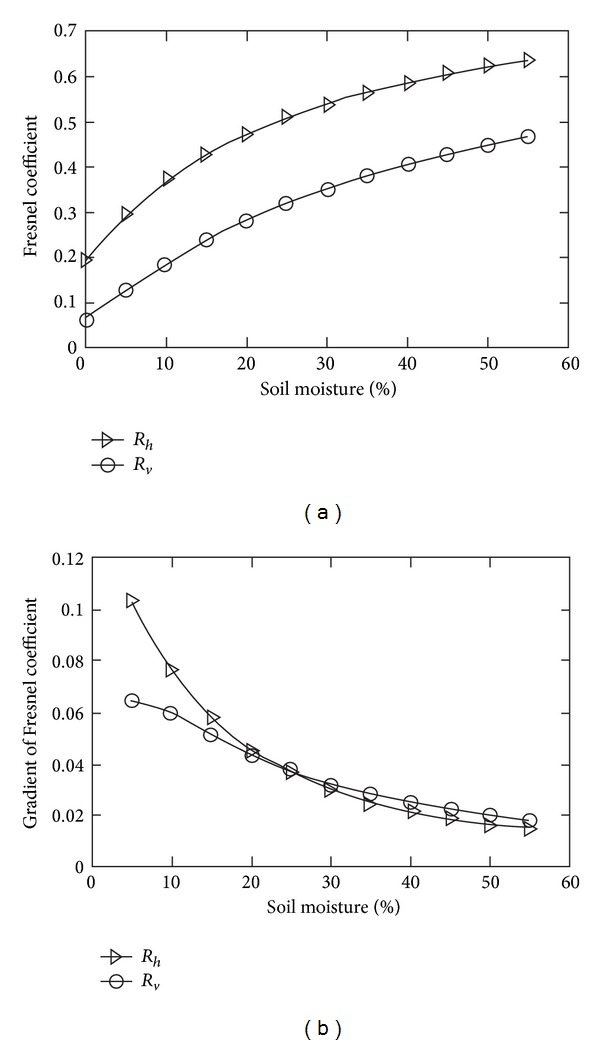
Relationship between the Fresnel coefficients and the gradient of the Fresnel coefficients with a variation in soil moisture.

To interpret this phenomenon, a salinity content of 30‰ is used as an example to demonstrate the relationship between the Fresnel coefficient and soil moisture and the relationship between the Fresnel coefficient gradient and soil moisture shown in Figure 9. The Fresnel coefficients of H and V polarization both increase with increasing soil moisture. The gradient of the Fresnel coefficients sharply decreases with an increase in water content and the change rate of the Fresnel coefficients is close to zero when the water content is greater than 40%, which illustrates that the Fresnel coefficients change slowly as the water content increases and then reach some constant. If other conditions remain unchanged, the radar signal trends towards stable.

### 4.4. Analysis of the Backscattering Coefficient of the Laboratory Dielectric Constant

**Figure 10:**
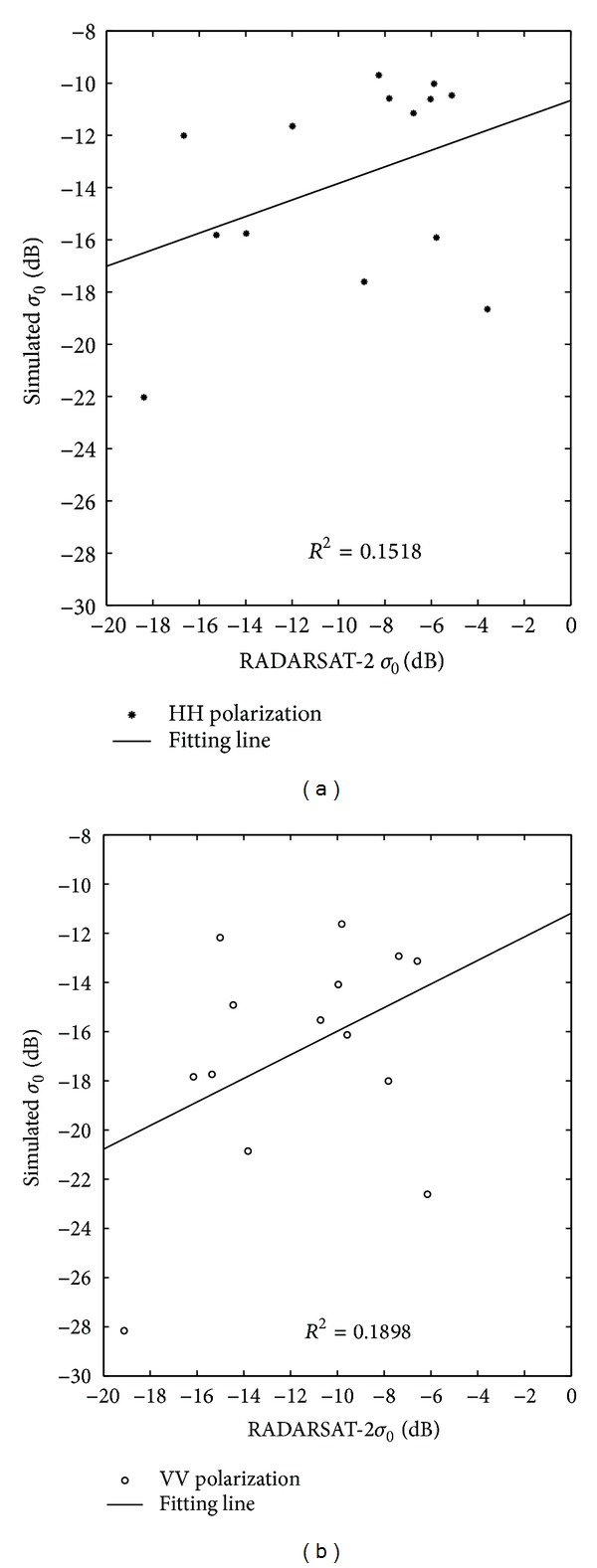
Simulated backscattering coefficient compared to the RADARSAT-2 images of the 16 ground samples, with HH polarization on (a) and VV polarization on (b).

**Figure 11:**
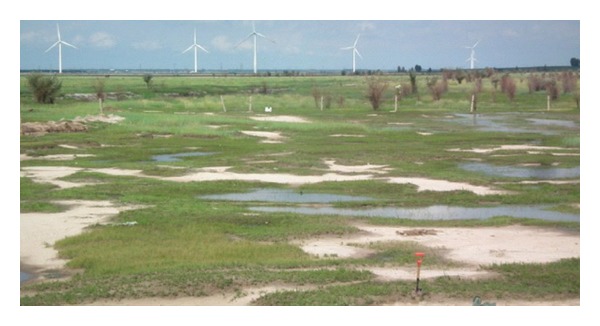
The landscape of saline-alkali soil in the research area.

The dielectric constants of samples were measured using an Agilent 83630A PNA Analyzer. The results of the measurement are analyzed in Section 4.2. The measured dielectric constant is an input parameter in the AIEM with an incident angle of 40° (the same for obtaining the radar image). The roughness parameter of the RMS height (*s*) is 0.27 cm, and the correlation length (*l*) is 3.5 cm, which are the average values for the research area. The backscattering coefficients with HH and VV polarization were derived using the AIEM with the input parameter described. For the same time period, the backscattering coefficients of the samples in the RADARSAT-2 images were extracted according to the latitude and longitude coordinates of the samples. The backscattering coefficient comparisons of model simulation results and image values of the samples with HH and VV polarization are shown in Figure 10 ((a) for HH polarization and (b) for VV polarization). The relationship between the simulated and *σ*_0_ image shown in Figure 10 and the correlation coefficient is 0.1518 and 0.1898, respectively. The relevancy was very poor for the model value and image value, which may be an influence of mixed pixels. The type of saline-alkali soil in this region is in a meadow, characterized by grass with areas of bare soil.  Figure 11 shows the landscape of the research area, which shows grass and bare soil distributed randomly, resulting in mixed pixels.

To improve the effect of grass in a pixel, the semiempirical water-cloud model used as a vegetation effect correction model was imported into the correction of the soil backscattering coefficient.

**Figure 12:**
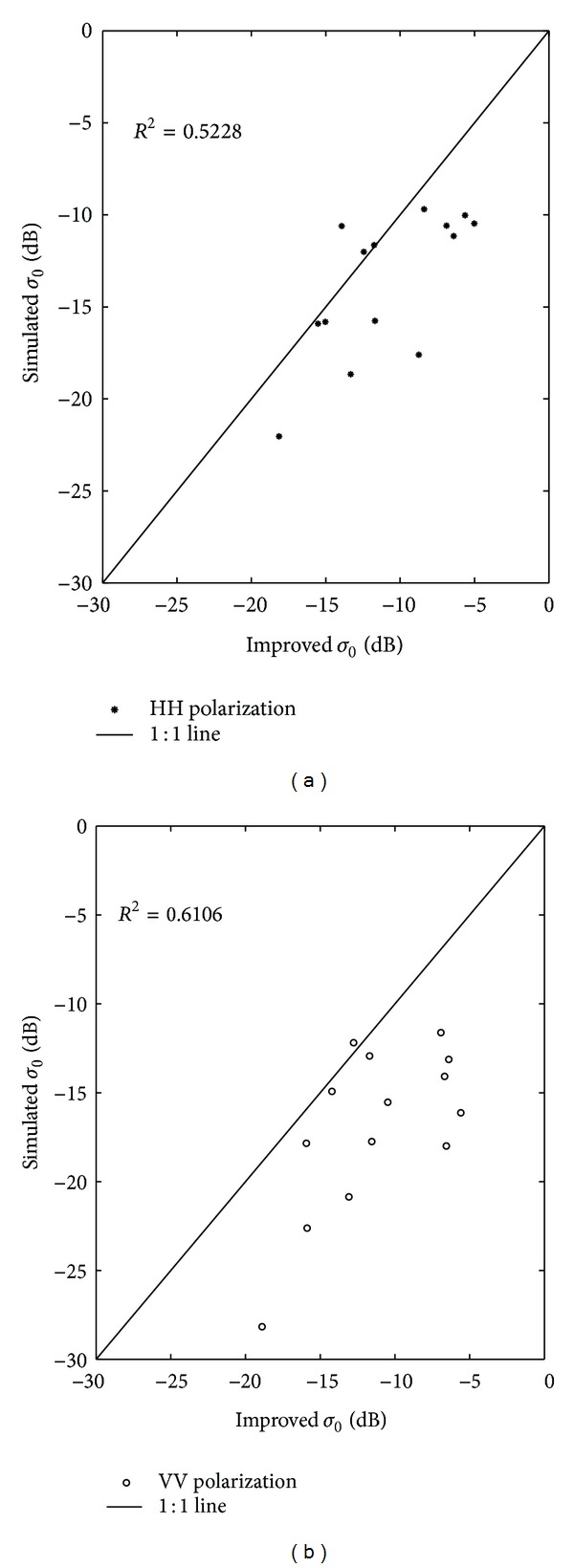
The improved backscattering coefficient from the water-cloud model compared with the simulated backscattering coefficient of the 16 ground samples, with HH polarization on (a) and VV polarization on (b).

The water-cloud model is described as

(3) $$\begin{array}{c} \sigma_{\text{con}}^{0}\left(\theta \right)=\sigma_{\text{veg}}^{0}\left(\theta \right)+\gamma^{2}\left(\theta \right)\sigma_{\text{soil}}^{0}\left(\theta \right), \end{array}$$

where *σ*_veg_^0^(*θ*) = *A*cos⁡(*θ*)[1 − *γ* ^2^(*θ*)], *γ* ^2^(*θ*) = exp⁡[−2*Bm*_*v*_/cos⁡(*θ*)], and *m*_*v*_ is the vegetation water content, which can be derived from optical images. The parameters of *A* and *B* are defined according to Table 3 [23]. The vegetation information improved the backscattering coefficient of ground soil samples. The results are shown in Figure 12. The correlation coefficients for the simulated *σ*_0_ (calculated from the laboratory-measured dielectric constant) and the improved *σ*_0_ (according to the water-cloud model) were 0.5228 and 0.6106, respectively, for the HH and VV polarization, which were both improved before. However, the simulated *σ*_0_ was higher than the *σ*_0_ value extracted from the image. The reason for the difference may be a reflection of the following two aspects. (1) The water-cloud model was based on two assumptions, the uniform distribution of scattering particles of the vegetation layer and the negligence of the multiple scattering between the vegetation layer and the soil surface; however, the grassland's interaction with bare, saline-alkali soil presented a very complicated landscape, which cannot be easily described by the water-cloud model. In other words, it was a problem of mixed pixels. (2) There may have been an error in the average values of surface roughness that we used for this region.

## 5. Conclusions

The complex dielectric constant for the salt-affected soil was analyzed using the modified Dobson dielectric mixing model. Then, the backscattering coefficients based on the simulated complex dielectric model were analyzed. Based on the analysis of the model's simulated data, laboratory measurements were performed on the ground samples. Then, the AIEM model was applied to the laboratory dielectric constants and compared with the backscattering coefficient extracted from radar imagery. The conclusions from the presented work are as follows.

1. The analysis of the simulated dielectric constant confirmed several dielectric properties of salt-affected soils. The first is that both the real and imaginary parts increase with the moisture content. The second is that salinity has little influence on the real part and *ε*′ decreases with increasing salinity, especially for soil with a high moisture content (*m*_*v*_ > 20%). The third is that the imaginary part *ε*′′ is strongly affected by both moisture and salinity. In particular, the higher the moisture content, the stronger the effect of salinity on the imaginary part.
2. The frequency effect on the salt-affected soil of laboratory measurement results paints a different picture for the simulated dielectric constant. The *ε*′′ sharply decreases at the low frequency range (0.5–3 GHz) and then exhibits an increasing trend form 5 GHz to 15 GHz, where the *ε*′′ becomes stable. This conclusion shows that the Dobson model combined with the seawater dielectric model is not suitable for soda saline-alkaline land.
3. The laboratory results of the frequency effect on the salinity also reveal that the combination of the C band and X band may be useful on the inversion of salinity and moisture.
4. The backscattering coefficients of VV and cross-polarization seem to be more sensitive than HH polarization on the salinity effect.
5. The saline-alkali lands in western Jilin Province are always mixed pixels with bare soil and grass as presented in the radar image, especially during the rainy season. The simple water-cloud model is not sufficient enough to solve the problem of mixed pixels.

Three important things must be done in the future. First, a suitable dielectric constant model for soda saline-alkali land is urgently needed. Second, the problem of mixed pixels needs to be solved through theoretical and technical support. Finally, polarization radar data has two important parameters, the amplitude and phase. The influence of salinity on the amplitude of the radar backscattering coefficient is initially considered, and the phase parameter, as a potential indicator for soil moisture detection, requires attention.

## Figures and Tables

**Table 1:** Characteristics of the SAR images in the study acquired by RADARSAT-2 sensors.

Parameter	RADARSAT-2 sensors
Frequency GHz (band)	5.405 (C band)
Wavelength (cm)	5.60
Polarization	HH/HV/VH/VV
Acquisition mode	Fine-Quad-Pol
Processing level	SLC (single look complex)
Resolution/m (rg/rz)	5.2∗7.6
Orbit	Ascending
Acquisition date	28/Jun/2013
Incidence angle interval	39.275°–40.975°

**Table 2:** Comparisons of Different Backscattering Coefficient Models.

Models	DM	SPM	POM	AIEM
Surface	All	Smooth	Rough	All
RMS (*s*) cm	0.3~3, *k* · *s* < 2.5	*k* · *s* < 0.3	*k* · *s* > 2	All
Correlation length (*l*)	—	*k* · *l* < 3, *s*/*l* < 0.3	*k* · *l* > 6, *l* ^2^ > 2.76 · *s* · *λ*	All
Incidence angle (*θ*)	30° ~ 65°	>35° ~ 40°	<35°	All
Frequency available (GHz)	1.5~11	Low Frequency	All	All

*λ* is the wavelength of electromagnetic wave, where *k* = 2*π*/*λ* is the wave-number.

**Table 3:** Values of vegetation parameters used in the semiempirical model.

Item	All vegetation	Rangelands	Cropland	Grassland
Parameter *A*	0.0012	0.0009	0.0018	0.0014
Parameter *B*	0.091	0.032	0.138	0.084
